# Supplementary material for: All-Organic PTFE Coated PVDF Composite Film Exhibiting Low Conduction Loss and High Breakdown Strength for Energy Storage Applications
Source: Polymers (Basel). 2023 Mar 5;15(5):1305. doi: 10.3390/polym15051305 (PMC10006870; doi:10.3390/polym15051305)
Supplement: Supplementary file 1 [file polymers-15-01305-s001.zip › polymers-2214210-supplementary.pdf]

Supporting Information

All-Organic PTFE Coated PVDF Composite Film Exhibiting Low Conduction Loss and High Breakdown Strength for Energy Storage Applications

Xiang-Shuo Meng, Yujiu Zhou *, Jianfeng Li, Hu Ye, Fujia Chen, Yuetao Zhao *, Qifeng Pan and Jianhua Xu

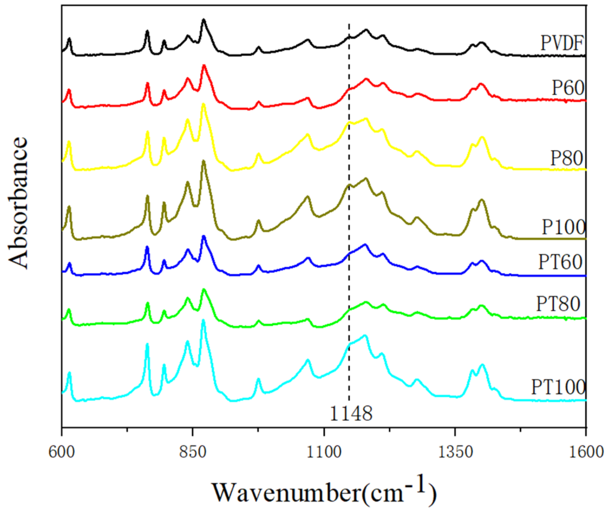

Figure S1. The FTIR spectra of PVDF and PTFE-c-PVDF.

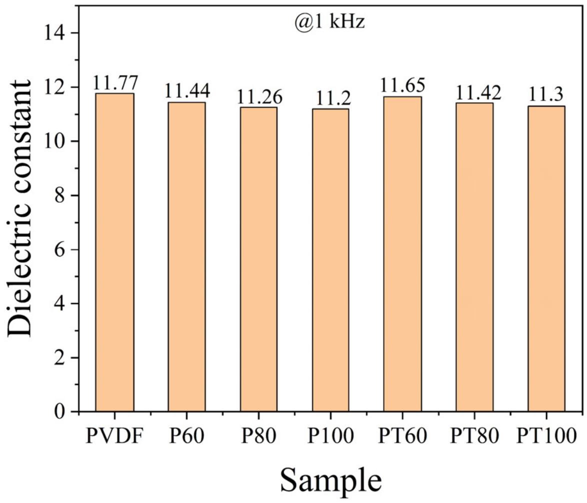

Figure S2. The dielectric constant of PVDF and its composite

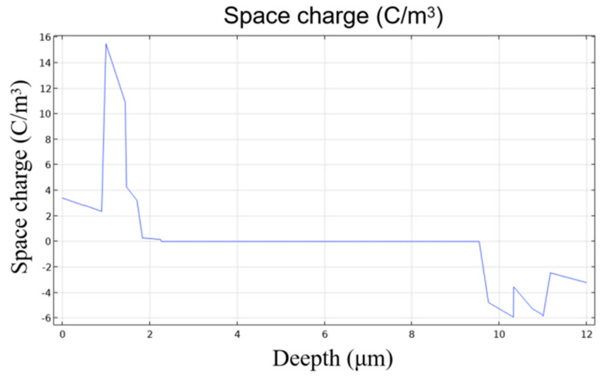

Figure S3. The Space charge simulation of PTFE-c-PVDF films

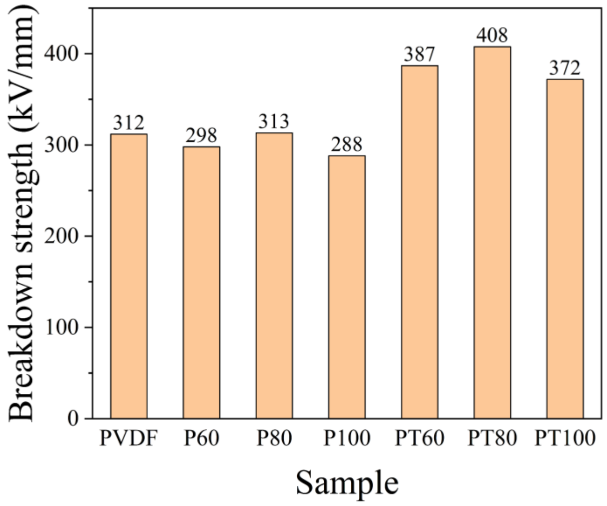

Figure S4. The breakdown strength of PVDF and its composite
